# Unraveling the Genetic Basis for the Rapid Diversification of Male Genitalia between *Drosophila* Species

**DOI:** 10.1093/molbev/msaa232

**Published:** 2020-09-15

**Authors:** Joanna F D Hagen, Cláudia C Mendes, Shamma R Booth, Javier Figueras Jimenez, Kentaro M Tanaka, Franziska A Franke, Luis Baudouin-Gonzalez, Amber M Ridgway, Saad Arif, Maria D S Nunes, Alistair P McGregor

**Affiliations:** Department of Biological and Medical Sciences, Oxford Brookes University, Oxford, United Kingdom; Department of Biological and Medical Sciences, Oxford Brookes University, Oxford, United Kingdom; Department of Biological and Medical Sciences, Oxford Brookes University, Oxford, United Kingdom; Department of Biological and Medical Sciences, Oxford Brookes University, Oxford, United Kingdom; Department of Biological and Medical Sciences, Oxford Brookes University, Oxford, United Kingdom; Department of Biological and Medical Sciences, Oxford Brookes University, Oxford, United Kingdom; Department of Biological and Medical Sciences, Oxford Brookes University, Oxford, United Kingdom; Department of Biological and Medical Sciences, Oxford Brookes University, Oxford, United Kingdom; Department of Biological and Medical Sciences, Oxford Brookes University, Oxford, United Kingdom; Centre for Functional Genomics, Oxford Brookes University, Oxford, United Kingdom; Department of Biological and Medical Sciences, Oxford Brookes University, Oxford, United Kingdom; Centre for Functional Genomics, Oxford Brookes University, Oxford, United Kingdom; Department of Biological and Medical Sciences, Oxford Brookes University, Oxford, United Kingdom; Centre for Functional Genomics, Oxford Brookes University, Oxford, United Kingdom

**Keywords:** evolution, development, *Drosophila*, sexual selection, morphology, gene regulatory networks

## Abstract

In the last 240,000 years, males of the *Drosophila simulans* species clade have evolved striking differences in the morphology of their epandrial posterior lobes and claspers (surstyli). These appendages are used for grasping the female during mating and so their divergence is most likely driven by sexual selection. Mapping studies indicate a highly polygenic and generally additive genetic basis for these morphological differences. However, we have limited understanding of the gene regulatory networks that control the development of genital structures and how they evolved to result in this rapid phenotypic diversification. Here, we used new *D. simulans*/*D. mauritiana* introgression lines on chromosome arm 3L to generate higher resolution maps of posterior lobe and clasper differences between these species. We then carried out RNA-seq on the developing genitalia of both species to identify the expressed genes and those that are differentially expressed between the two species. This allowed us to test the function of expressed positional candidates during genital development in *D. melanogaster*. We identified several new genes involved in the development and possibly the evolution of these genital structures, including the transcription factors Hairy and Grunge. Furthermore, we discovered that during clasper development Hairy negatively regulates *tartan* (*trn*), a gene known to contribute to divergence in clasper morphology. Taken together, our results provide new insights into the regulation of genital development and how this has evolved between species.

## Introduction

To understand the evolution of animal morphology, we need to better link genotypic and phenotypic changes. This requires identifying the causative genes, how they are integrated into gene regulatory networks, and how changes in these interactions alter developmental processes and consequently the phenotype (Stern 2011; Nunes et al. 2013; Kittelmann et al. 2018). There has been great progress in identifying genes that cause changes in animal morphology (reviewed in Martin and Orgogozo [2013]). However, we still lack information on the genes that contribute to changes in quantitative traits, such as organ size, and how they combine to achieve this.

**Fig. 1.**
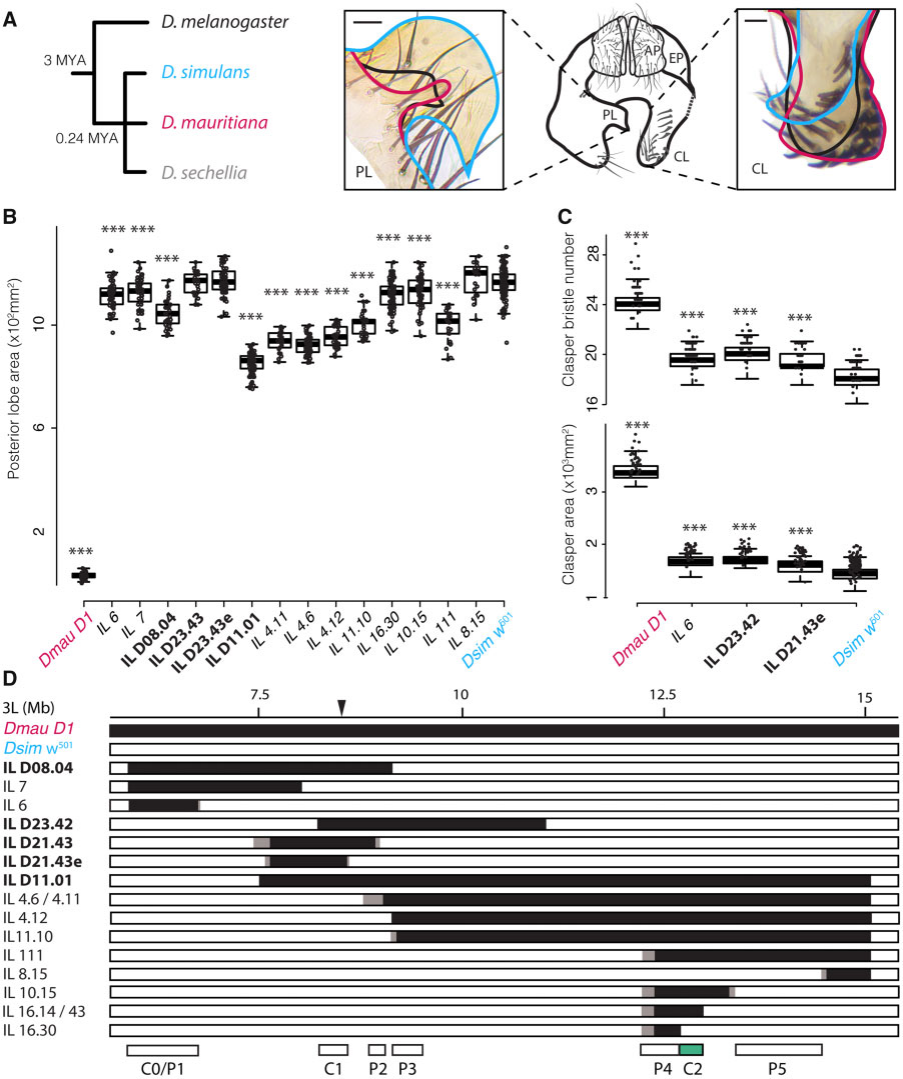
Differences in external male-terminal structures among *Drosophila melanogaster* subgroup species and introgression mapping between *D. simulans* and *D. mauritiana*. (*A*) Relationships of *D. melanogaster* and species of the *D. simulans* clade (left). The central diagram depicts a schematic of male genital arch morphology of *D. melanogaster* (posterior view). The posterior lobes (left-hand box) typically obscure visualization of the claspers (right-hand box), and therefore they are shown here dissected away on the right-hand side of the central schematic. The relative size and shape of the lobes and the claspers of *D. melanogaster* (black), *D. simulans* (blue), and *D. mauritiana* (red) are illustrated in the left and right schematics and images, respectively. *D. simulans* has much larger posterior lobes and smaller claspers, with fewer, thinner, and shorter bristles than *D. mauritiana* and *D. melanogaster*. Posterior lobes (PL), claspers (CL), anal plates (AP), and epandrium (EP). Scale bars = 20 µm. (*B–D*) Mapping and phenotypic effect of candidate regions on posterior lobe size (*B*), clasper bristle number (*C*, upper plot), and clasper area (*C*, lower plot). Boxes indicate the range, upper and lower quartiles, and median for each sample. Asterisks indicate significant comparisons with *Dsim w*^501^ where *P* < 0.001*** (Dunnett’s test for posterior lobe and clasper size, Dunn’s test for clasper bristle number, supplementary file 2*D*–*F*, Supplementary Material online). Differences in the effect of the introgressed regions (supplementary file 3 and supportive text, Supplementary Material online) on posterior lobe size (*B*) and clasper size/bristle number (*C*), allowed refinement of candidate regions P1–P5, C0, and C1 (*D*). The previously identified C2 region is shown in green (Hagen et al. 2019) (*D*). Black bars indicate *Dmau D1* DNA, white bars indicate *Dsim w*^501^ DNA, and gray boxes regions containing break points that have not been precisely determined. The black triangle indicates the position of P-element insertion originally used for generating the introgressions. New introgression lines are shown in nonbold font.

The size and shape of male genital organs evolve rapidly among species, driven by sexual selection (Eberhard 1985, 2010; Hosken and Stockley 2004; House et al. 2013; Simmons 2014). For example, the epandrial posterior lobes and claspers (surstyli) have changed dramatically in size in the *Drosophila simulans* species clade in the last 240,000 years (Garrigan et al. 2012) (fig. 1*A*). Both the claspers and posterior lobes play important roles during copulation. The claspers open the female oviscapt through interdigitization of bristles, and help achieve correct copulatory positioning (Robertson 1988; Acebes et al. 2003; Jagadeeshan and Singh 2006; Kamimura and Mitsumoto 2011; Yassin and Orgogozo 2013; Masly and Kamimura 2014; Mattei et al. 2015), whereas the posterior lobes also contribute to stability during mating by inserting into grooves on the female tergites (Robertson 1988; Kamimura and Mitsumoto 2011; Yassin and Orgogozo 2013).

The posterior lobes are a novelty of the *D. melanogaster* species subgroup (Kopp and True 2002; Jagadeeshan and Singh 2006; Glassford et al. 2015). In *D. mauritiana*, they are small, thin, finger-like projections in comparison to the much larger, helmet-shaped lobes of *D. simulans* (fig. 1*A*). *D. melanogaster* has intermediate-sized lobes, which are trapezoid shaped (fig. 1*A*), whereas the *D. sechellia* lobes are also intermediate in size and resemble “boots.” It is important to note that there is some variation within species but the extremes of intraspecific variation do not overlap with the differences observed between species (McNeil et al. 2011; Hackett et al. 2016).

The claspers lie beneath the posterior lobes, and about twice as large in *D. mauritiana* compared with *D. simulans*, with a third more bristles (True et al. 1997; Tanaka et al. 2015) (fig. 1*A*). The morphology of these bristles also differs between the species, with the *D. mauritiana* bristles being generally shorter and thicker than those of *D. simulans* (True et al. 1997; Tanaka et al. 2015). *D. sechellia* male claspers have very similar morphology to those of *D. simulans*, whereas the claspers of *D. melanogaster* appear to be intermediate between *D. mauritiana*, and *D. simulans*/*D. sechellia* (fig. 1*A*).

Genetic mapping of changes to posterior lobe and clasper morphology among *D. melanogaster* subgroup species have shown that these differences are polygenic and generally additive (Coyne et al. 1991; Liu et al. 1996; Laurie et al. 1997; True et al. 1997; Macdonald and Goldstein 1999; Zeng et al. 2000; Tanaka et al. 2015, 2018). For example, up to 19 QTL have been identified for the difference in posterior lobe size between *D. mauritiana* and *D. simulans*, and QTL have been mapped to all major autosomal arms for the differences in clasper size between these species (Laurie et al. 1997; True et al. 1997; Zeng et al. 2000; Tanaka et al. 2015, 2018). Therefore, it appears that many loci contribute to these differences in genital organ size.

We previously used an introgression-based approach to fine-scale map QTL on chromosome arm 3L underlying posterior lobe and clasper size differences between *D. mauritiana* and *D. simulans* (True et al. 1997; Zeng et al. 2000; Tanaka et al. 2015; Hagen et al. 2019). The genomes of these lines were *D. simulans*, apart from introgressed regions of *D. mauritiana* DNA on 3L (Tanaka et al. 2015; Hagen et al. 2019). The regions that we found to contribute to posterior lobe and clasper size differences were mutually exclusive; suggesting that different genes underlie divergence in these two structures (Tanaka et al. 2015). Furthermore, this approach revealed that sequence divergence in *tartan* (*trn*), which encodes a leucine-rich repeat transmembrane protein, contributes to the larger claspers of *D. mauritiana* compared with *D. simulans* (Hagen et al. 2019). This is likely due to more extensive and persistent expression of *trn* in the developing claspers in *D. mauritiana* (Hagen et al. 2019). However, since *trn* does not appear to contribute to posterior lobe size differences and explains only 16% of the clasper size difference between the species (Hagen et al. 2019), there must be additional loci involved in posterior lobe and clasper size differences on chromosome arm 3L.

To try to identify other causative genes on 3L, we generated new introgression lines to further refine existing candidate regions (Tanaka et al. 2015). We complemented this approach with RNA-seq on the developing genitalia of both species to identify genes expressed and differentially expressed both genome-wide and in the mapped regions. Subsequent functional testing of positional and expression candidate genes in *D. melanogaster* identified novel players involved in genital development, including the transcription factors (TFs) Grunge (Gug) and Hairy (H), which appear to positively and negatively regulate clasper size, respectively. Furthermore, we found that H represses *trn* expression in the developing claspers suggesting that changes in this regulatory interaction may contribute to interspecific differences in this structure. Taken together our findings provide new insights into the genetic interactions that underlie genital development, as well as the divergence of genital morphology between *Drosophila* species.

## Results

### Mapping Genomic Regions Underlying Male Genital Divergence between *D. simulans* and *D. mauritiana*

Previously, we resolved the C2 candidate region for clasper size divergence between *D. simulans and D. mauritiana* by successfully identifying *trn* as the causative gene in this region (Hagen et al. 2019). In order to increase the resolution of other candidate regions contributing to male genitalia divergence (Tanaka et al. 2015), we generated 23 new introgression lines with smaller introgressed *D. mauritiana* regions in a *D. simulans* background (fig. 1*B–D* and supplementary file 1, Supplementary Material online). We mapped clasper size and clasper bristle number to two regions that collectively explain 16.8% of clasper size differences between these species (table 1 and supplementary supportive text, Supplementary Material online). We confirmed the location and effect size of the previously identified C1 region (Tanaka et al. 2015) and identified a new region, C0, which explains 11% of the divergence in clasper morphology (fig. 1*D*, table 1, and supplementary supportive text, Supplementary Material online). We mapped posterior lobe size to five regions that collectively explain 29.3% of posterior lobe size differences between *D. mauritiana* and *D. simulan*s, two of which, P4 and P5, are new (fig. 1*D*, table 1, and supplementary supportive text, Supplementary Material online). In total, these regions contain 380 protein-coding genes (as annotated in *D. melanogaster*, table 1).

**Table 1.** Summary of Candidate Regions Underlying Clasper and Posterior Lobe Divergence.

Candidate Region	Phenotypic Effect Size^a^ (%)	Number of Expressed Genes						
		Total Number of Genes^b^	Total	Diff^c^	Upregulated^c^		Genes Tested by RNAi in *D. mel*	
					*Dsim w* ^501^	*Dmau D1*	Total	Developmental Candidates^d^
C0	11 (20)	99	69	14	5	9	7	*sgl*
C1	6 (21)	58	35	6	4	2	32	*hairy*, *Cpr66D*, *Gug*, *Mcm7*, *foi*
P1	4	99	69	14	5	9	7	*—*
P2	6	7	2	1	1	0	7	—
P3	6	71	49	10	5	5	5	—
P4	5	52	38	5	3	2	2	—
P5	9	93	67	13	7	6	0	—

a The phenotypic effect size is calculated as a percentage of the difference in phenotype between the parental strains. Brackets in C regions indicate effect size for clasper bristle number.

b Protein-coding orthologs in *Drosophila melanogaster R6.24*.

c Differentially expressed between *Dmau D1* and *Dsim w*^501^, *P*_adj_ (FDR) < 0.05.

d Genes that significantly affect either clasper size, bristle number, or posterior lobe size compared with both UAS and driver controls (*P* < 0.05) after RNAi knock-down are considered developmental candidates.

### Analysis of Genes Expressed in Developing Male Genitalia

We next carried out RNA-seq on stages 2 and 4.5 of male genital development in *D. mauritiana* strain D1 (*Dmau D1*) and *D. simulans* strain *w*^501^ (*Dsim w*^501^) (Hagen et al. 2019). This allowed us to assay the genes expressed in the developing genitalia and those differentially expressed between these two species genome-wide and in our mapped regions.

We detected expression of 8,984 and 8,458 genes above the threshold value of 1 transcripts per million (TPM) in all biological replicates in the developing genital arches of *Dsim w*^501^ and *Dmau D1*, respectively (supplementary file 4*A*, Supplementary Material online). A total of 760 genes are only expressed in *Dsim w*^501^, whereas 264 genes are only expressed in *Dmau D1*. However, many of these genes (114 and 121 genes, respectively) have low expression in the species where they are detected (<2 TPM on an average between replicates) and therefore are less likely to underlie functional expression differences between species. Gene ontology (GO) analysis of the remaining 676 detected genes in *Dsim w*^501^ indicated the most significant enrichment is in genes involved in heme binding (supplementary file 4*B*, Supplementary Material online) such as *Cyp4d14*, *Cyp9b2*, *Cyp6d5*, *Cyp6t1*, *Cyp4g1*, *Cyp12a5*, *Cyt-c-d*, *Cyp6d2*, *glob2*, *Cyp6a20*, and *Cyp4aa1*. The remaining 143 detected genes exclusive to *Dmau D1* were enriched for ion transmembrane transporters (supplementary file 4*B*, Supplementary Material online), the majority of which were ionotropic receptors (IRs) (*IR76b*, *IR7g*, *IR60b*, *IR7f*, *IR25a*) as well as the ionotropic glutamate receptor *eye-enriched kainate receptor* (*Ekar*).

Of the 8,194 genes detected in both species, 1,169 were significantly differentially expressed between *Dsim w*^501^ and *Dmau D1*, with 547 upregulated in the former and 622 in the latter, respectively (*P*_adj_ < 0.05, supplementary file 4*C*, Supplementary Material online). Using the Kyoto Encyclopedia of Genes and Genomes (KEGG), we determined that 14/547 and 16/622 of these differentially expressed genes encode proteins in signaling pathways (supplementary file 4*D*, Supplementary Material online). This includes components of the mTOR, Notch, Hippo, Toll, and Imd pathways that are upregulated in *Dsim w*^501^, and members of the mTOR, MAPK, Wnt, FOX0, Toll, and Imd pathways that are upregulated in *Dmau D1* (supplementary file 4*D*, Supplementary Material online). However, note that none of these genes is located in the introgressed regions we have analyzed.

To further explore divergence in gene regulation in the developing male genitalia of these species, we next assessed the expression of TF-encoding genes. We found 802 out of 994 genes-encoding TFs and cofactors are expressed in the developing genitalia of *Dmau D1* and *Dsim w*^501^ according to our RNA-seq data set (supplementary file 4*E*, Supplementary Material online). We identified eight TF genes that appear to be exclusively expressed in the developing male genitalia of *Dmau D1*, whereas 16 appear to be exclusive to *Dsim w*^501^. However, three and ten of these TFs, respectively, were detected at relatively low levels (TPM < 2) and are therefore not likely to contribute to functional regulatory differences in genital development between species. Of the 778 TF genes expressed in both species (supplementary file 4*E*, Supplementary Material online), 49 are differentially expressed with 33 upregulated in *Dmau D1*, and 16 upregulated in *Dsim w*^501^ (supplementary fig. 1, Supplementary Material online). This includes five of the TF genes whose spatial expression in the developing genitalia of *D. melanogaster* was recently characterized (*hinge3*, *Myb oncogene-like*, *single stranded-binding protein c31A*, *Sox21b* and *enhancer of split m3*, *helix-loop-helix*) (Vincent et al. 2019).

We then focused on which of the genes in our mapped introgression regions are expressed in the developing genitalia. We found that 260 of the 380 protein-coding genes in the introgression-mapped regions could be detected in our RNA-seq data, including 31 TFs (supplementary file 5, Supplementary Material online). 49 of the expressed candidate genes are differentially expressed between *Dsim w*^501^ and *Dmau D1*, with about half the genes being upregulated in each (table 1). This includes one TF that is upregulated in *Dsim w*^501^ (*mirror* [P4]) and four in *Dmau D1* (*meiotic central spindle*, *Sox21b*, *CG17359* [all P5], and *CG10147* [C0/P1]).

### Identifying Developmental Candidate Genes

We next sought to test if the positional candidate genes that are expressed in the genitalia according to our RNA-seq data have a role in the development of either the posterior lobes or the claspers. To do this, we performed RNAi in *D. melanogaster* to knockdown candidate genes in the smallest posterior lobe (P2) and clasper (C1) candidate regions, as well as a selection of promising genes from the other regions based on their expression profiles (table 1 and supplementary files 5 and 6, Supplementary Material online). RNAi knockdown of the two expressed genes within P2 had no significant effect on posterior lobe size (nor on clasper size) (supplementary file 6, Supplementary Material online).

**Fig. 2.**
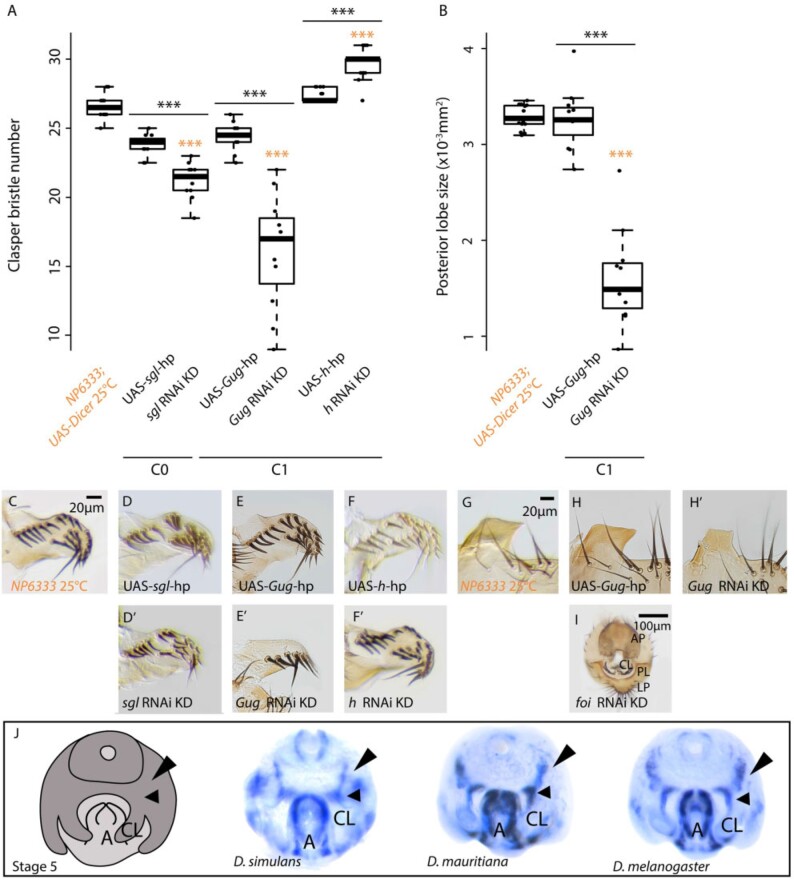
Functional analysis of positional candidate genes in *D. melanogaster* male genitalia. (*A*) Knocking down C0 candidate gene *sgl* (Tanaka et al. 2015) and C1 candidate gene *Gug* resulted in significantly fewer clasper bristles compared with both the UAS-*Gug*-hp (black asterisks, *P* < 0.001) and *NP6333* driver controls (orange asterisks, *P* < 0.001). In contrast, knocking down C1 candidate gene *h* resulted in significantly more clasper bristles compared with the *NP6333* driver (*P* < 0.001, orange asterisks) and UAS-*h-*hp controls (*P* < 0.001, black lines and black asterisks). (*B*) In addition, knocking down C1 candidate gene *Gug* resulted in the development of significantly smaller posterior lobes compared with the UAS-*Gug*-hp (orange asterisks) and *NP6333* driver controls (*P* < 0.001, black asterisks). Asterisks indicate significant differences detected with Tukey’s pairwise comparisons, where *P* < 0.001*** and *P* > 0.05 = “ns” (supplementary file 6, Supplementary Material online). Orange indicates comparisons between the *NP6333* driver control and UAS-gene-hp controls/gene knockdowns, whereas comparisons between UAS controls and knockdowns are indicated by black lines and black asterisks. Boxes indicate the range, upper and lower quartiles, and median for each sample. hp, hairpin; KD, knockdown. (*C*–*I*) Morphology of claspers and posterior lobes in *NP6333* driver controls, UAS controls, and gene knockdowns (*D*–*F*′ and *H*, *H*′). (*J*) An illustration of stage 5 male genitalia (excluding the posterior lobes) and in situ hybridizations of *Cpr66D* in *Dsim w*^501^, *Dmau D1*, and *Dmel w*^1118^. *Cpr66D* transcripts were detected in a wider domain along the clasper inner edge (small arrowheads) and in bands extending toward the anal plates (large arrowheads) in the two species with larger clasper. *Crpr66D* is also expressed in the aedeagus of all three species. CL, clasper primordia; A, aedeagus (internal genitalia). Note that *sgl* RNAi knockdown data were generated in Tanaka et al. (2015) and reanalysed here.

In combination with our previous study (Tanaka et al. 2015), we have now carried out RNAi for all expressed C1 candidate genes with available UAS lines (32 out of 35, supplementary file 6, Supplementary Material online). We previously observed that RNAi knockdown of *cuticular protein 66D* (*Cpr66D*) and *minichromosome maintenance 7* (*Mcm7*) results in larger and smaller claspers, respectively (Tanaka et al. 2015). In addition to these two genes, we have now found that knocking down *hairy* (*h*), *Grunge* (*Gug*), and *fear of intimacy* (*foi*) significantly affects clasper bristle number and clasper morphology (fig. 2*A*, *E*′, *F*′, and *I*; supplementary file 6, Supplementary Material online). Knockdown of *h* results in larger claspers with more bristles (fig. 2*A*, *F* and *F*′), whereas reducing *Gug* expression gives rise to smaller claspers with fewer bristles (fig. 2*A*, *E* and *E*′). This implies that the H and Gug TFs play opposite roles in the regulation of clasper size. Interestingly, *Gug* also appears to positively regulate posterior lobe size; since knocking down this gene significantly reduces the size of these structures (fig. 2*B*, *H* and *H*′; supplementary file 6, Supplementary Material online). *foi* knockdown results in severe developmental defects, with fusion of the appendages of the male external genitalia including the claspers (fig. 2*I*).

**Fig. 3.**
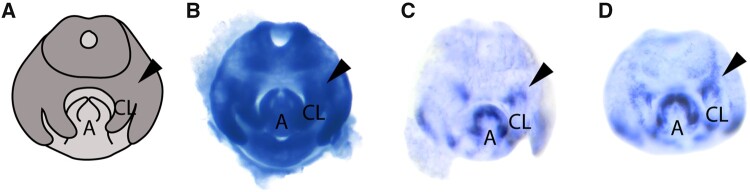
*trn* expression expands in the clasper primordia upon *h* RNAi knockdown in *D. melanogaster*. (*A*) A schematic of genital development based on DAPI at stage 5 (Hagen et al. 2019). (*B*) *h* mRNA in situ hybridization in *D. melanogaster w*^1118^. (*C*) *trn* mRNA in situ hybridization on UAS parental control and (*D*) *trn* mRNA in situ hybridization upon *h* RNAi knockdown at stage 5. Both *trn* in situ hybridizations were carried out in parallel and allowed to develop for the same length of time. *h* RNAi results in a distortion of *trn* expression at the base of the claspers compared with the UAS control (black arrows). CL, claspers; A, aedeagus. *N* = 5 for each experiment.

Therefore, the C1 region contains five promising clasper developmental candidate genes. However, none of these genes is differentially expressed between *Dsim w*^501^ and *Dmau D1* (upregulated in the latter, supplementary file 5, Supplementary Material online). Analysis of the spatial expression of *Cpr66D* during genital development revealed that this gene is expressed in a wider domain along the inner clasper edge in *D. melanogaster* and *D. mauritiana* compared with *D. simulans*, and in bands extending from this region toward the lateral edge of the anal plates (fig. 2*J*).

Region C0/P1 encompasses 99 genes (table 1). About 69 of these genes are expressed in the developing genitalia according to our RNA-seq data, with 14 exhibiting significantly differential expression between *Dmau D1* and *Dsim w*^501^ (table 1 and supplementary file 5, Supplementary Material online). Together with our previous study (Tanaka et al. 2015), we have now carried out RNAi against two of these differentially expressed genes (*SP1173* and *CG9953*), and five other nondifferentially expressed genes (*sugarless* [*sgl*], *CG32388*, *ventral veins lacking* [*vvl*], *CG10064*, and *lactate dehydrogenase* [*ImpL3*]) in addition to *sugarless* [*sgl*] which we had previously knocked down (Tanaka et al. 2015). Only RNAi against *sgl* produced a phenotype (Tanaka et al. 2015). *sgl* appears to have a role in clasper development because RNAi knockdown of this gene led to significantly smaller claspers, but had no effect on the posterior lobes (fig. 2*A*, *D*, and *D*′; supplementary file 6, Supplementary Material online) (as shown previously in Tanaka et al. 2015).

### Interactions between Genes Underlying Clasper Divergence

*trn* is the only gene identified so far that has been shown to contribute to clasper differences between *D. simulans* and *D. mauritiana*. The *D. mauritiana* allele of *trn* generates larger claspers with more bristles than the *D. simulans* allele (Hagen et al. 2019). This is likely achieved through the expanded and/or more enduring expression of *trn* in the developing claspers in *D. mauritiana* compared with *D. simulans* (Hagen et al. 2019). It was previously shown that the transcriptional corepressor *h*, represses *trn* expression during embryogenesis in *D. melanogaster* (Chang et al. 1993; Kok et al. 2015) and in *Drosophila* Kc cells (Bianchi-Frias et al. 2004). Since we found that *h* RNAi in *D. melanogaster* results in significantly larger claspers with more bristles (fig. 2), we hypothesized that this gene might negatively regulate clasper size through repression of *trn*.

Consistent with a previous study (Vincent et al. 2019), we found that *h* is ubiquitously expressed throughout the male genitalia of *D. melanogaster*, including the clasper primordia (fig. 3*B*). We then analyzed the expression of *trn* in the developing genitalia of *h* RNAi knockdowns (fig. 3*C* and *D*; supplementary fig. 2, Supplementary Material online). Upon *h* RNAi knockdown *trn* expression at the base of the developing claspers at 46 hAPF/stage 5 appears to be expanded and the edges of the domain are less well defined compared with controls, with the bands of expression extending in wisps dorsally (fig. 3*C* and *D*; supplementary fig. 2, Supplementary Material online). The extreme differences in *trn* expression upon *h* RNAi are shown in supplementary figure 2, Supplementary Material online, and may explain the range of clasper bristle count data generated by this treatment (fig. 2*A* and supplementary file 6, Supplementary Material online). This ectopic expression indicates that the larger claspers produced upon *h* RNAi knockdown in *D. melanogaster* are likely due to increased *trn* expression at the base of the claspers, and that *h* acts upstream of *trn* in the clasper GRN. Despite being ubiquitously expressed throughout the male genitalia (fig. 3*B*), the selective targeting of *trn* by H may explain the role of this TF in regulating clasper but not posterior lobe development; since *trn* is not expressed in the developing posterior lobes (fig. 3*C*). However, *h* is expressed throughout the genital arch, and so its role in the development of other genital structures is still unclear.

## Discussion

### Regions on Chromosome Arm 3L Contributing to Inter- and Intraspecific Variation in Posterior Lobe and Clasper Size

As found previously, all regions identified through our introgression approach affect the claspers and/or posterior lobes consistently in the direction of their differences between the two species: *Dmau D1* DNA resulted in larger claspers and smaller posterior lobes than *Dsim w*^501^ and vice versa (Zeng et al. 2000; Tanaka et al. 2015; Hagen et al. 2019).

Also consistent with previous studies, we have shown clasper area and clasper bristle number map to the same genomic locations, which suggests that the same genes may influence both traits (Tanaka et al. 2015; Hagen et al. 2019). This could at least in part be explained by the process of bristle formation through lateral inhibition (Heitzler and Simpson 1991) and consequently large claspers developing more bristles than small claspers. It is not clear, therefore, whether selection drove changes in clasper bristle number, and clasper size changed as a by-product, or vice versa. However, the interdigitization of clasper bristles with those of the female oviscapt would perhaps argue for the former scenario (Mattei et al. 2015).

Apart from C0/P1, all regions identified only affected either the claspers or the posterior lobes, which suggests different genes underlie the diversification in size of these two structures between *D. simulans* and *D. mauritiana* (fig. 1). The effects observed for C0/P1 could be explained by a single evolved locus that is able to affect growth of the claspers and posterior lobes in opposite directions with *D. mauritiana* C0/P1 alleles generating smaller posterior lobes and larger claspers (fig. 1*B* and *C*). Alternatively, since C0/P1 is still a relatively large region, it is possible that further mapping would resolve this region into distinct clasper and posterior lobe loci.

Interestingly, genes within region C0/P1 may underlie intraspecific variation as well as interspecific differences in posterior lobe size. This region overlaps with the 3L QTL peak observed in other interspecific mapping studies of differences in posterior lobe size between *D. simulans* and *D. mauritiana* or *D. sechellia* (Liu et al. 1996; Macdonald and Goldstein 1999; Zeng et al. 2000; Masly et al. 2011), as well as QTL peaks found in studies that mapped genetic variation underlying differences in posterior lobe size between *D. melanogaster* strains (McNeil et al. 2011; Takahara and Takahashi 2015; Hackett et al. 2016). Several other studies have also found cases where intraspecific variation maps to the same genomic region as interspecific variation (Nuzhdin and Reiwitch 2000; Gleason et al. 2002; Tatsuta and Takano-Shimizu 2006). Therefore, P1 genes represent excellent candidates for contributing to variation in the size of this structure both within and between species.

### Genome-Wide Gene Expression During Genital Development in *D. mauritiana* and *D. simulans*

We carried out RNA-seq to identify and compare genes expressed in the developing genitalia between *D. mauritiana* and *D. simulans*. We were able to filter out positional candidates and also obtain a genome-wide perspective of gene activity during genital development as well as inferring differential expression between species.

In both species, all the key genes known to pattern the genital disc are expressed, such as homeotic genes and sex-determination genes (Casares et al. 1997; Chen and Baker 1997; Estrada and Sanchez-Herrero 2001; Keisman and Baker 2001; Sanchez and Guerrero 2001; Estrada et al. 2003) and signaling genes, like *wingless*, *decapentaplegic*, and *hedgehog* (Casares et al. 1997; Chen and Baker 1997; Abdelilah-Seyfried et al. 2000; Keisman and Baker 2001; Sanchez and Guerrero 2001). We also detected expression of 80% TF-encoding genes, including those previously shown to pattern the male genital disc, such as *cubitus interruptus*, *engrailed* (Kornberg et al. 1985; Eaton and Kornberg 1990; Simmons and Garcia-Gonzalez 2011), *dachshund* (Keisman and Baker 2001), *distal-less* (Estrada and Sanchez-Herrero 2001), and *Drop* (Chatterjee et al. 2011).

We found that a small proportion of genes (<10%) are exclusively expressed in the developing genitalia of either *Dsim w*^501^ or *Dmau D1*. The *Dsim w*^501^ male genital-specific genes are enriched for iron ion binding proteins, whereas the *Dmau D1* genes are enriched for multiple IRs. IRs are a conserved family of chemosensory receptors best known for their role in olfaction (Benton et al. 2009; Grosjean et al. 2011; Silbering et al. 2011; Min et al. 2013; Ziegler et al. 2013). Interestingly, some IRs, for example, *IR52c* and *IR52d*, are candidate taste and pheromone receptors (Koh et al. 2014) expressed in a sexually dimorphic manner on the sensilla of the *D. melanogaster* male foreleg, which makes contact with the female during courtship (Koh et al. 2014). The neurons in which these IRs are expressed in *D. melanogaster* males are only activated upon contact with females of the same species (Koh et al. 2014). Therefore, the striking differences in IR expression between male *Drosophila* species’ genitalia may be an evolved mechanism to prevent conspecific mating.

Of the genes that are expressed in both *Dsim w*^501^ and *Dmau D1*, we found that 1,169 were differentially expressed. This includes 30 signaling pathway components and 49 TF-encoding genes. This suggests that the regulatory landscape of developing genitalia is generally conserved between *D. mauritiana* and *D. simulans*. However, the differentially expressed TFs will help to better understand the gene regulatory networks involved in genital development and evolution, and represent excellent candidate genes for further investigation.

### Functional Analysis of Expressed Positional Candidates on Chromosome 3L During Genital Development

We have now analyzed the function of 57 of the expressed genes by RNAi knockdown in *D. melanogaster*, including 32 out of the 35 genes expressed in C1 (including those we studied previously in Tanaka et al. [2015]), as well as all expressed genes in P2 (table 1). Note that we did not just focus on differentially expressed genes because genes can exhibit localized differences in expression during genital development that may contribute to morphological differences (Hagen et al. 2019).

RNAi against the expressed P2 genes did not have any significant effect on the posterior lobes (supplementary file 6, Supplementary Material online). RNAi against some of these genes simply may not have worked for various reasons, including when partial knock-down of the gene may not be sufficient to result in a phenotype (although numerous P2 genes were tested with multiple RNAi constructs, supplementary file 6, Supplementary Material online). Given this caveat of potential false negatives from RNAi, this approach allows us to prioritize genes for downstream analysis rather than completely exclude them as candidates. It remains possible that a nonprotein coding element in region P2 may explain the phenotypic effect of this region on posterior lobe size. Indeed, P2 encompasses a microRNA, mir-4940, as well as a long noncoding RNA CR45408 (Thurmond et al. 2019). Therefore, the causative element in P2 could be either of these factors, or a long-range enhancer responsible for the differential regulation of a gene outside P2 between these two species.

Our functional analysis of region C0/P1 identified one excellent candidate gene, *sgl*. *sgl* has been implicated in boundary formation and may interact with Wnt signaling (Hacker et al. 1997). RNAi against *sgl* resulted in smaller claspers (fig. 2*A* and *D*′; Tanaka et al. 2015), but this gene is not differentially expressed between *D. mauritiana* and *D. simulans*. However, since C0/P1 is a large region that is likely to contain many other developmental candidates, higher resolution mapping, and functional analysis of genes in C0/P1 is needed.

RNAi against C1 genes revealed five interesting genes for clasper development and evolution: *Gug*, *foi*, *Mcm7*, *Cpr66D*, and *h* (this study; Tanaka et al. 2015). *Cpr66D* expression is more extensive along the inner edge of the claspers and in bands extending toward the anal plates in *Dmau D1* compared with *Dsim w*^501^ (fig. 2*K*). *Cpr66D* encodes a structural protein that forms chitin-based cuticle (Ren et al. 2005; Chandran et al. 2014; Stahl et al. 2017) and its role in genital development merits further study.

We also found evidence for potential interactions between other genes in mapped regions during genital development. Repression of *trn* by H has been predicted (Bianchi-Frias et al. 2004; Kok et al. 2015) or shown (Chang et al. 1993) in different developmental contexts. We found that H also negatively regulates *trn* expression in the developing claspers of *D. melanogaster*; with larger claspers generated by *h* RNAi likely being caused by expansion of the *trn* expression domain (figs. 2*A* and *F*′ and 3*D*; supplementary fig. 2, Supplementary Material online). H also negatively regulates *trn* expression during embryogenesis to help define compartmental boundaries (Chang et al. 1993; Pare et al. 2019). Therefore, this regulatory interaction could represent a more general mechanism for coordinating the correct positioning of cells during development. However, *h* is not differentially expressed between *Dsim w*^501^ and *Dmau D1* and appears to be ubiquitously expressed in the developing genitalia of *D. melanogaster* (fig. 3*B*) (Vincent et al. 2019). Although it is possible that there could also be localized differences in *h* expression in the developing genitalia, these observations suggest that the differences in *trn* expression between *Dsim w*^501^ and *Dmau D1* could be the result of protein-coding changes that affect the DNA-binding efficiency of H, or variation in the number and/or sensitivity of H binding sites in *trn* regulatory elements. Indeed, there are several predicted H binding sites across the *trn* locus, but identification of *trn* genital enhancers and further analyses of H binding sites between *D. mauritiana* and *D. simulans* is needed to test this further.

In addition to *trn*, H may regulate multiple genes during clasper development including candidates revealed by our mapping and functional analyses. For example, H is also predicted to negatively regulate the C1 candidate gene, *Gug* (Yeung et al. 2017). Indeed, Gug itself is predicted to regulate the C0 candidate gene *sgl*, as this gene contains a Gug binding site in its intron (Yeung et al. 2017). However, since Gug acts as a transcriptional corepressor, and RNAi against both *Gug* and *sgl* reduces clasper size, it is unclear at this stage if there is a regulatory interaction between these genes in the developing claspers. It will be interesting to test these predictions in the future to learn more about the architecture of the gene regulatory network for clasper development and how this evolved during the rapid diversification of these structures.

## Materials and Methods

### Introgression Line Generation and Phenotyping

We generated new recombinants in our candidate regions by backcrossing virgin *IL D11.01/Dsim w*^501^ heterozygous females, and virgin *IL D08.04*/*Dsim w*^501^ heterozygous females to *Dsim w*^501^ males. *IL D1101* is an introgression line with *D. mauritiana D1* DNA in the genomic location 3L: 7527144.15084689 Mb and encompasses the candidate regions C1, P2, and P3 (Tanaka et al. 2015). *IL D08.04* is an introgression line with *D. mauritiana w*^−^ DNA on 3L: 5911371.9167745 Mb (R2.02 *D. simulans*) and includes candidate regions P1 and C1 (Tanaka et al. 2015). New recombinants were detected by selecting for the loss of the visible marker D1 (True et al. 1997; Tanaka et al. 2015) (fig. 1*D*), restriction fragment length polymorphisms, and sequencing markers (see supplementary file 7, Supplementary Material online for primer list). New introgression lines (supplementary file 1, Supplementary Material online) were all maintained as homozygous stocks.

Male genitalia were phenotyped from flies cultured under controlled growth conditions. All males used were progeny of ten females and five males that were transferred every 2 days, and allowed to develop at 25 °C in a 12-h light–12-h dark cycle incubator unless otherwise stated. All adult males were maintained on a standard cornmeal diet at 25 °C for at least 3 days before collection and storage in 70% EtOH.

Where possible, two or three replicates of ILs were phenotyped. Replicates are defined as introgression lines derived from the same recombination event and therefore containing the same introgressed region of *D. mauritiana* DNA. The abdominal tip and T1 leg were dissected for each fly in 70% EtOH, and transferred to Hoyer’s medium. Using entomological pins, the posterior lobes were then dissected away from the claspers and anal plates. The claspers, posterior lobes, and T1 tibia were mounted in Hoyer’s medium for imaging.

Images were taken using a Zeiss Axioplan light microscope at 250× magnification for the claspers and lobes and 160× for the T1 tibia, using a DFC300 camera. Clasper area, posterior lobe size, and tibia length were measured manually using ImageJ (Schneider et al. 2012), and bristle number was counted for each clasper (supplementary file 2*A*, Supplementary Material online). T1 tibia length was used as a proxy for body size, in order to assess consistency in rearing conditions and to ensure genital differences were not a result of general differences in size. Most introgression lines showed no significant difference in T1 tibia length compared with *Dsim w*^501^ (supplementary file 2*G*, Supplementary Material online), and since genitalia are hypoallometric (Coyne et al. 1991; Liu et al. 1996; Macdonald and Goldstein 1999; Eberhard 2009; Shingleton et al. 2009; Masly et al. 2011), the phenotypic data were not corrected for body size. A detailed description of statistical methods and the comparisons used to map candidate regions based on these data can be found in the supplementary supportive text, Supplementary Material online.

### RNA Sequencing and Differential Expression Analysis

Three independent RNA-seq library replicates were generated for *Dsim w*^501^ and *Dmau D1* developing male genitalia. Flies were reared under the above conditions, and white prepupae collected. Males were selected using gonad size and allowed to develop in a humid container at 25 °C until either stage 2 or stage 4.5 (Hagen et al. 2019). Between these stages, the claspers develop from a ridge structure to a distinct appendage separate from the surrounding tissue, and the posterior lobe has begun to extend outward from the lateral plate primordia (Hagen et al. 2019). The anterior of pupae were impaled with a needle onto a charcoal agar plate and submerged in 1×PBS. Dissection scissors were used to remove the distal tip of the pupal case and the outer membrane, and pressure applied to the abdomen to allow the developing genitalia to be quickly expelled from the pupal case and dissected away from the abdomen. Note that the entire genital arch, including internal genital organs (but not including abdominal tissue), was isolated for RNA extraction. The genitalia from 15 males from each stage were collected and then combined in TRIzol (ThermoScientific). RNA was then extracted using standard procedures. Quality and quantity of RNA were verified using a Qubit fluorometer. Samples were sequenced by the NERC Biomolecular Analysis Facility (NBAF) at the Centre for Genomic Research, University of Liverpool, where dual-indexed, strand-specific RNA-seq libraries were prepared using NEBNext polyA selection and Ultra Directional RNA preparation kits. Samples were then sequenced using Illumina HiSeq 4000 (paired-end, 2×150-bp sequencing). These RNA-seq data have been deposited in the ArrayExpress database at EMBL-EBI (www.ebi.ac.uk/arrayexpress) under accession number E-MTAB-9465 (https://www.ebi.ac.uk/arrayexpress/experiments/E-MTAB-9465). Ribosomal reads were filtered out using default settings in SortMeRNA version 4.2.0 (Kopylova et al. 2012), and Trimmomatic version 0.38 (Bolger et al. 2014) was used to trim low-quality reads using default parameters. The remaining *D. simulans* reads were mapped against the reannotated transcriptomes of *D. simulans*, “GSE76252_ReanDsim_with_ ReanDmau_GeneSet_1to1orth,” and the remaining *D. mauritiana* reads were mapped against the reannotated transcriptome of *D. mauritiana*, “GSE76252_PubDmau_ with_ReanDsim_exoutput_1to1orth” (Torres-Oliva et al. 2016), using Bowtie2 version 2.3.5 with the –very-sensitive-local option (Langmead and Salzberg 2012). Reads mapped to reannotated genomes with an overall alignment rate of 68–69%. The SAM files were then converted to BAM files, sorted by coordinate, and index files created using Samtools version 1.10 (Li et al. 2009). Duplicate reads were marked but left in the data set. These data were then used with HTSeq-count version 0.11.1 in order to generate raw read counts for each gene (Anders et al. 2015). TPM was calculated using these counts in order to quantify gene expression, and the DEseq2 R package version 1.28.1 was used to determine differential expression between species using the default parameters (Love et al. 2014). Genes were considered to be expressed if TPM > 1 in all three biological replicates. Genes were only considered differentially expressed in comparisons where *P*_adj_ (FDR) < 0.05.

### Gene Ontology Analysis

In order to investigate the nature of the expressed, not expressed, and differentially expressed genes in our RNA-seq data set, we determined their ontology using PANTHER version 15.0 (Thomas et al. 2003). We conducted overrepresentation tests (released 09/11/2019) of GO (released December 9, 2019) for the positional genes against the *D. melanogaster* reference list using the Fisher test (Thomas et al. 2006). Genes were considered significantly overrepresented when *P*_adj_ (FDR) < 0.05.

### Pathway Database Analysis

To identify potential differences in signaling pathway gene expression between *Dsim w*^501^ and *Dmau D1* developing male genitalia, we searched for differentially expressed genes in the KEGG pathway database (Kanehisa and Goto 2000). Those annotated as signaling pathway components are reported in supplementary file 4*D*, Supplementary Material online.

### Annotation of TFs Present in RNA-Seq Data

In order to extract the genes encoding TFs from the RNA-seq data set, we used the databases of genes from Flymine (https://www.flymine.org/flymine/begin.do;Lyne et al. 2007), amiGO (http://amigo.geneontology.org/;Carbon et al. 2009), and Flybase (Thurmond et al. 2019), and bioinformatic analysis and manual curation from Hens et al. (2011). We filtered the genes in our data set corresponding to TFs by their GO terms and gene groups in molecular function using the previously mentioned sources. The GO terms used were the following: “FlyTF_putativeTFs” from Flymine (Lyne et al. 2007), “transcription factor regulator activity” and “transcription factor coregulator activity” from amiGO (Carbon et al. 2009), “transcription factor gene group” and “transcription coregulator activity” from Flybase (Thurmond et al. 2019) and the data set of TFs from Hens et al. (2011). Genes that were annotated with these terms in any of the four resources were considered TF genes and used for downstream analysis.

### RNAi Knockdown of Candidate Genes

The developmental role of genes was tested using RNAi in *D. melanogaster*. UAS-RNAi lines for these genes were provided by the Vienna *Drosophila* RNAi Center and the Bloomington *Drosophila* Stock Center (see supplementary file 6, Supplementary Material online for stock numbers). UAS males of candidate genes were crossed to *NP6333–Gal4* (“*NP6333*”) driver virgins (*P[GawB]PenNP6333*) (Chatterjee et al. 2011) carrying *UAS-Dicer-2 P[UAS-Dcr-2.D]* (Stieper et al. 2008). RNAi knockdown was conducted at either 25 or 28 °C (supplementary file 6, Supplementary Material online) (Tanaka et al. 2015), under identical rearing conditions, and dissection, imaging, and analysis were carried out as described above (supplementary file 6, Supplementary Material online). To assess the role of a gene during genitalia development, we compared the phenotype of genital structures of gene knockdowns against the respective *NP6333* driver controls using a Dunnett’s test (supplementary file 6, Supplementary Material online). If the gene knockdown phenotype differed significantly from the *NP6333* driver control, we then assessed whether or not this significant effect is a result of genetic background (e.g., an effect of the UAS-parental phenotype), or reflects a role of the gene in genital development. To do this, we compared all three experimental groups of males using an ANOVA (supplementary file 6, Supplementary Material online). If this was significant, we then analyzed where these differences arise from using a Tukey’s test, and only concluded genes have a developmental role in the genitalia if the RNAi knockdown males were significantly different in phenotype compared with both parental controls.

### In Situ Hybridization

Sample collection, RNA extraction, cDNA synthesis, and probe synthesis were conducted as described in Hagen et al. (2019). We performed in situ hybridization to detect expression of *Cpr66D* in *D. mauritiana*, *D. simulans*, and *D. melanogaster*, *h* in *Dmel w*^1118^ and *trn* in *UAS-h* Bloomington TRiP 27738, *NP6333-Gal4*; *UAS-Dicer* x *UAS-h* Bloomington TRiP 27738 using species-specific probes. Probes were generated using the following oligos (forward followed by reverse) with the addition of T7 linker sequences added to the 5′ end of each primer; *trn* (514 bp) ATCGAGGAGCTGAATCTGGG and TCCAGGTTACCATTGTCGCT (Hagen et al. 2019), *Cpr66D* (314 bp) CTCCTCGTATCAGTTTGGCTTC and CTGGTGGTACT GTGGCTGCT. Antisense *h* probes were generated by amplification using T7 primers from a BLUESCRIBE plasmid that contained sequences for all three *h* coding exons (a gift from B. Jennings, Oxford Brookes University). In situ hybridizations were based on the Carroll lab “Drosophila abdominal in situ” protocol (http://carroll.molbio.wisc.edu/methods.html) with minor modifications. All in situ hybridizations were conducted at least twice, with *n* = 5–10 in each experiment.

## Supplementary Material

Supplementary data are available at *Molecular Biology and Evolution* online.

## Supplementary Material

msaa232_Supplementary_Data

## Data Availability

The data underlying this article have been deposited in the ArrayExpress database at EMBL-EBI (www.ebi.ac.uk/arrayexpress) under accession number E-MTAB-9465 (https://www.ebi.ac.uk/arrayexpress/experiments/E-MTAB-9465) or are available in its Supplementary Material online.
