# Correction to: Unraveling the Genetic Basis for the Rapid Diversification of Male Genitalia between *Drosophila* Species

**DOI:** 10.1093/molbev/msac207

**Published:** 2022-10-18

This is a correction to: Joanna F D Hagen, Cláudia C Mendes, Shamma R Booth, Javier Figueras Jimenez, Kentaro M Tanaka, Franziska A Franke, Luis Baudouin-Gonzalez, Amber M Ridgway, Saad Arif, Maria D S Nunes, Alistair P McGregor, Unraveling the Genetic Basis for the Rapid Diversification of Male Genitalia between *Drosophila* Species, *Molecular Biology and Evolution*, Volume 38, Issue 2, February 2021, Pages 437–448, https://doi.org/10.1093/molbev/msaa232

In the originally published version of this paper, the authors included re-analysis of phenotypic data from RNAi knockdown of genes reported in their previous publication, Tanaka et al. 2015 (Genetics, doi: 10.1534/genetics.114.174045). The authors have since discovered an error in one of the RNAi lines used, which meant that they actually targeted the gene *Sulf1* on chromosome arm 3R instead of *Surf1* on chromosome arm 3L as reported.

The paper has been corrected to remove these data for *Sulf1* from the text of the paper, Figure 2 and supplementary files 5 and 6. The authors have also further clarified the genes that were first tested in Tanaka et al., (2015) and those data reanalysed here in the context of new mapping and gene expression data.

