# Supplementary material for: Unraveling the Genetic Basis for the Rapid Diversification of Male Genitalia between Drosophila Species
Source: Mol Biol Evol. 2020 Sep 15;38(2):437–48. doi: 10.1093/molbev/msaa232 (PMC7826188; doi:10.1093/molbev/msaa232)
Supplement: msaa232_Supplementary_Data [file msaa232_supplementary_data.zip › msaa232_Supplementary_Data/msaa232-suppl_data/Supplementary Figure 1.docx]

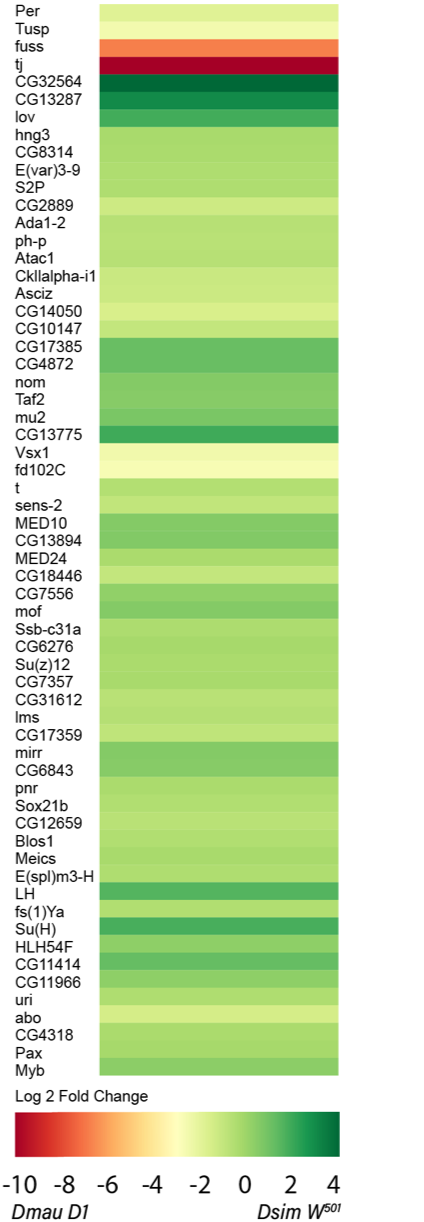


**Supplementary Figure 1. Differentially expressed transcription factors**

Transcription factors expressed (TPM > 1) differentially (padj FDR < 0.05) between *Dmau D1* and *Dsim w*^501^ shown with a Log 2 fold change scale.
