# Supplementary material for: Unraveling the Genetic Basis for the Rapid Diversification of Male Genitalia between Drosophila Species
Source: Mol Biol Evol. 2020 Sep 15;38(2):437–48. doi: 10.1093/molbev/msaa232 (PMC7826188; doi:10.1093/molbev/msaa232)
Supplement: msaa232_Supplementary_Data [file msaa232_supplementary_data.zip › msaa232_Supplementary_Data/msaa232-suppl_data/Supplementary Figure 2.docx]

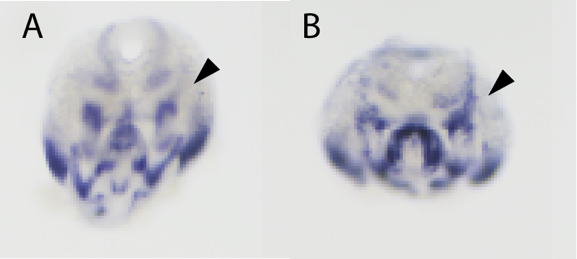


**Supplementary Figure 2. Extreme affects of *h* RNAi knockdown on *trn* expression**

A and B show the weakest and strongest affects of *h* RNAi knockdown on *trn* expression, respectively. The most subtle effect detected was a weak expansion of the *trn* expression domain at the base of the developing claspers (black arrow, A) compared to the more striking expansion of this expression domain when the effect was strongest (black arrow, B). *trn* mRNA in situ hybridisation was conducted in *D. melanogaster* *w*^1118^ at stage 5 (Hagen et al. 2019).
